# Competition and drought affect cleistogamy in a non-additive way in the annual ruderal *Lamium amplexicaule*

**DOI:** 10.1093/aobpla/plae036

**Published:** 2024-06-24

**Authors:** Bojana Stojanova, Anežka Eliášová, Tomáš Tureček

**Affiliations:** Department of Biology and Ecology, Faculty of Science, University of Ostrava, Ostrava, Chittussiho 10, 71000Czech Republic; Department of Biology, University Federico II of Naples, Via Vicinale Cupa Cintia, 21, 80126 Naples, Italy; Department of Biology and Ecology, Faculty of Science, University of Ostrava, Ostrava, Chittussiho 10, 71000Czech Republic; Department of Biology and Ecology, Faculty of Science, University of Ostrava, Ostrava, Chittussiho 10, 71000Czech Republic

**Keywords:** cleistogamy, drought, ecotype, habitat succession, interspecific competition, *Lamium amplexicaule*, mixed-mating, phenotypic plasticity, resource limitation

## Abstract

Competition affects mixed-mating strategies by limiting available abiotic or biotic resources such as nutrients, water, space, or pollinators. Cleistogamous species produce closed (cleistogamous, CL), obligately selfed, simultaneously with open (chasmogamous, CH), potentially outcrossed flowers. The effects of intraspecific competition on fitness and cleistogamy variation can range from limiting the production of costly CH flowers because of resource limitation, to favouring CH production because of fitness advantages of outcrossed, CH offspring. Moreover, the effects of competition can be altered when it co-occurs with other environmental variations. We grew plants from seven populations of the ruderal *Lamium amplexicaule*, originating from different climates and habitats, in a common garden experiment combining drought, interspecific competition, and seasonal variation. All these parameters have been shown to influence the degree of cleistogamy in the species on their own. In spring, competition and drought negatively impacted fitness, but the CL proportion only increased when plants were exposed to both treatments combined. We did not observe the same results in autumn, which can be due to non-adaptive phenotypic variation, or to differences in soil compactness between seasons. The observed responses are largely due to phenotypic plasticity, but we also observed phenotypic differentiation between populations for morphological, phenological, and cleistogamy traits, pointing to the existence of different ecotypes. Our data do not support the hypothesis that CL proportion should decrease when resources are scarce, as plants with reduced growth had relatively low CL proportions. We propose that variation in cleistogamy could be an adaptation to pollinator abundance, or to environment-dependent fitness differences between offspring of selfed and outcrossed seeds, two hypotheses worth further investigation. This opens exciting new possibilities for the study of the maintenance of mixed-mating systems using cleistogamous species as models that combine the effects of inbreeding and reproductive costs.

## Teaser

*Lamium amplexicaule* is a cleistogamous plant and can adjust its production of open and closed flowers in response to interspecific competition and drought. While both factors decrease plant size and total reproductive output, their combined effects are non-additive. Moreover, the production of cheap and reliable closed flowers increases when plants are exposed to drought and competition simultaneously, but not to watering and competition, despite plants being the same size in both treatment combinations. The switch from closed to open flower production in *Lamium amplexicaule* is likely dependent on regulatory factors that are not linked to plant size but are triggered by environmental cues signalling conditions favourable for the production of different flower types.

## Introduction

About half of all flowering plants reproduce through a mixture of self-pollination (selfing) and cross-pollination (outcrossing), i.e. they have mixed-mating systems (Whitehead *et al.* 2018). The maintenance of mixed-mating is still an evolutionary enigma with no universal explanation (Goodwillie *et al.* 2005), but theoretical and empirical observations show that mixed-mating can be maintained when individuals are exposed to environmental variation, with different environments favouring different mating strategies (Lloyd 1979, 1992; Lloyd and Schoen 1992; Cheptou and Mathias 2001; Kalisz *et al.* 2004; Morgan and Wilson 2005; Barrett and Harder 2017).

Selfing is considered a more economical means of reproduction—as it foregoes pollinators for effective pollination and fertilization, selfing requires less pollen and no specific floral structures to attract pollinators (selfing syndrome, Sicard & Lenhard, 2011). Moreover, parents transmit more gene copies to offspring through facultative selfing compared to strict outcrossing (automatic advantage of selfing, Fisher 1941). Selfing could thus be the preferred mating strategy of plants in environments that impede plant growth through lack of resources or otherwise stressful conditions, or where pollinators and mating partners are scarce (Kalisz *et al.* 2004; Jorgensen and Arathi 2013; Arathi and Smith 2023). This is only possible if selfed offspring do not suffer from high inbreeding depression which cancels out its advantages (e.g. > 50% decrease in performance of selfed relative to outcrossed offspring completely cancels out the automatic advantage, Lande and Schemske 1985).

Competition can affect the mating system by imposing limitations to abiotic (water, nutrients, space), as well as biotic (pollinators) resource availability. Moreover, competition can differently affect individuals with different levels of inbreeding. All these factors could interact and result in counter-intuitive outcomes of competition for mating systems. For example, although competitive environments are unfavourable for plant growth, taxa with competitive ecological strategies (CSR; competition-stress tolerant-ruderal ecological strategy, sensu Grime 1977) generally have low selfing rates (Munoz *et al.* 2016). Such trends have also been observed at ecological scales, for example, populations of *Crepis sancta* growing in more competitive, late-succession habitats, have reduced ability for selfing and reduced selfing rates, compared to populations growing in non-competitive, early succession habitats (Cheptou *et al.* 2002). Moreover, the effects of competition can be exacerbated or diminished when co-occurring with other types of environmental variation. In various horticultural species, combining interspecific competition and drought stress can increase or decrease flower production depending on the specific treatment, although the fitness consequences of these changes were not evaluated (Blanusa *et al.* 2009).

Cleistogamy is the ability that some plants have to produce closed (CL), obligately selfing flowers. Most cleistogamous species also produce open (chasmogamous—CH), showy, and potentially outcrossed flowers, often within the same plant (dimorphic cleistogamy, sensu Lord 1981). Cleistogamous species can thus be considered to have a mixed-mating system provided that their CH flowers reliably outcross. In dimorphic cleistogamous species the proportion of CL flowers is often plastic and can vary in response to environmental cues such as photoperiod, temperature, drought, herbivory, or intraspecific competition (Schemske 1978; Bell and Quinn 1987; Schmitt and Ehrhardt 1987; Le Corff 1993; Stojanova *et al.* 2016; Seguí *et al.* 2018; Heywood and Smith 2021). While the effects of intraspecific competition have been studied, either on their own (Waller 1980) or in association with other biotic factors (Steets *et al.* 2006), the effects of interspecific competition on the degree of cleistogamy have not been explicitly investigated so far.

Several explanations have been proposed for the maintenance of plastic cleistogamy, taking into account the contrasting reproductive (selfing vs outcrossing) and non-reproductive (cost of flower production, pollination efficiency) features of the two floral types. CL flowers are more economical to produce and more reliably pollinated, resulting in a fitness advantage of CL relative to CH offspring ranging from 15% to 231% (Oakley *et al.* 2007). CH flowers, on the other hand, can produce less inbred and genetically more diverse offspring, which could withstand a wider and more unpredictable range of environmental conditions (Waller 1980). Two classic explanations taking these features into account are that CL flowers should be favoured when resources are scarce (Schemske 1978), or when pollinator activity is low and unreliable (Kalisz and Vogler 2003). Numerous studies show that the relative CL production increases in resource poor environments or in response to environmental cues that indicate unfavourable conditions for growth or pollination (Lord 1982; Berg and Redbo-torstensson 1998; Cheplick 2007; Oakley *et al.* 2007; Albert *et al.* 2011; Veena and Nampy 2019; Soto *et al.* 2024). In the annual cleistogamous *Lamium amplexicaule* it has been observed that although total flower production is reduced under competition, the CH proportion is higher in natural populations growing in more competitive, late succession habitats which limit plant growth, despite climatic differences between populations (Stojanova *et al.* 2014). An alternative explanation for the maintenance of plastic cleistogamy is that the genetically more diverse and less inbred CH offspring are better suited to survive unfavourable conditions, as observed in some non-cleistogamous species (Cheptou *et al.* 2000). Therefore, the relative production of CH flowers will increase in environments which induce high inbreeding depression, or environments with intense and unpredictable selective pressures which require larger adaptive potential of the population (Stojanova *et al.* 2014, 2020; Koontz *et al.* 2017). Even when inbreeding depression is low in cleistogamous species, there are intrinsic fitness advantages specific to CL and CH offspring that could help maintain plastic cleistogamy in variable environments (Trapp and Hendrix 1988; Culley 2000; Munguía-Rosas *et al.* 2013; Ansaldi *et al.* 2019; Stojanova *et al.* 2020).

In this study we assess the effect of interspecific competition and drought stress in spring and autumn on the variation of cleistogamy and fitness in *L. amplexicaule* using a full-factorial, common garden experiment combining competition, drought, and seasonal variation to (i) assess the effects of these environmental factors on cleistogamy and to (ii) test whether the observed variation in natural populations is due to genetic differentiation or phenotypic plasticity. In line with Stojanova *et al* (2014) previous observations of cleistogamy in natural populations, we expect that competition alone, despite having negative effects on plant growth and flower production, would decrease the CL proportion, whereas the effects of competition are less predictable when combined with drought and seasonal variation. Moreover, we predict a strong plastic response of cleistogamy to environmental variation but also population differentiation between early and late succession habitats. Understanding how the CL and CH production and other traits in cleistogamous species vary in response to competition is the necessary first step in the study of the adaptive character of plastic cleistogamy in complex, variable biotic and abiotic environments.

## Materials and Methods

### Model species

*Lamium amplexicaule* is an annual cleistogamous species. The species starts flowering by producing exclusively CL flowers (constitutive cleistogamy, Lord 1979), and then switches to producing CH and CL flowers in variable proportions in response to environmental cues (plastic cleistogamy). The CL proportion in *L. amplexicaule* can vary in response to cues such as photoperiod, temperature, competition, or seasonal variation, increasing up to 100% in cold, short, autumn days, and decreasing down to 50% in warm, long, spring days (Lord 1982; Stojanova *et al.* 2014, 2016). *Lamium amplexicaule* has a ruderal ecological strategy (Grime 1977). The species is the first to colonize and thrives in regularly disturbed soils such as vineyards and early tilled crop fields before any other vegetation develops (further called early succession habitats). Plants in early succession habitats develop several dozens of flowering stems bearing hundreds of flowers. Occasionally, *L. amplexicaule* populations can establish and persist in habitats that have been colonized by other vegetation, such as field margins or lawns with undisturbed soil (late succession habitats). These populations have reduced size, their individuals produce fewer, elongated stems, bearing a few dozen flowers in total. Observations of four natural populations showed that while *L. amplexicaule* populations in early succession habitats produce overall more flowers per stem, and have higher CL proportions compared to populations growing in late succession habitats. Moreover, even though CH flowers have lower fertilization success in early compared to late succession habitats, the population outcrossing rate of CH flowers was estimated to be 25% across different habitats and climates, suggesting that CH flowers consistently outcross although at relatively low rates (Stojanova *et al.* 2014).

*Lamium amplexicaule* populations can have two different life cycles based on their germination timing—spring and winter annuals. Spring annuals germinate in late winter and start flowering as early as six to eight weeks after germination. In temperate climates, spring annuals can produce an additional autumn cohort that germinates at the end of summer and flowers by mid-autumn. Winter annuals germinate in autumn and spend the winter in a vegetative state, then flower at approximately the same time as spring annuals (Baskin and Baskin 1981; Stojanova *et al.* 2016).

### Plant populations and seed material

In the spring of 2018, a total of eight *L. amplexicaule* populations were sampled in Bohemian and Silesian Czechia (temperate climate), North Macedonia (Mediterranean), and Israel (desert, Table 1). Whenever possible, we sampled two populations at nearby locations in two different habitats, early and late succession. At the end of the flowering season (from March to May, depending on the country), 16–36 plants per population (total of 244) with ripe seeds were collected and left to dry in paper bags at room temperature. Seeds were collected and stored in paper bags in a dark, dry place at room temperature until the beginning of the experiment.

**Table 1.** Characteristics of the studied populations. Country: NMK—Macedonia, CZ—Czech Republic, IL—Israel. N maternal plants—number of maternal plants sampled in the field. Toponym—nearest geographical toponym (placename) to the sampling site. The GPS coordinates of CZ2 were not recorded at the time of seed collection.

Pop ID	Country	Succession stage	GPS coordinates	Geographic toponym	N maternal plants
NMK1	NMK	Late	41°23ʹ07.84″N 22°12ʹ57.53″E	Ljubaš	36
NMK2	NMK	Late	41°23ʹ06.47″N 22°12ʹ55.81″E	Demir Kapija	16
NMK3	NMK	Early	41°23ʹ07.84″N 22°12ʹ57.53″E	Demir Kapija	25
IL1	IL	Early	33°06ʹ47.52″N 35°31ʹ58.73″E	Tel Kedes	19
CZ1	CZ	Late	50°03ʹ19.14″N 14°17ʹ10.84″E	Zličín	16
CZ2	CZ	Early	NA	Kraví Hora	25
CZ3	CZ	Late	50°46ʹ47.08″N 7°11ʹ0.545″E	Ostrava Hl N	16
CZ4	CZ	Early	50°03ʹ27.71″N 14°16ʹ17.27″E	Zličín	20

### Experimental design

All experimental work was conducted at the Botanical Garden of the University of Ostrava (49°49ʹ39.1″N, 18°19ʹ32.7″E, Table 1).

On the 20th of March 2019, at least 50 mg of seeds from each population were sown in a 1 L pot filled with peat substrate (AGRO CS a.s. Říkov, Czech Republic). Population NMK1 was not included in the spring experiment because it did not germinate. On the 8th of April 2019, up to 50 seedlings per population were transplanted in individual planters filled with peat substrate and placed in a greenhouse. On the 19th of April 2019, when the root system of the seedlings was sufficiently developed, up to 36 seedlings per population were transplanted directly into the soil in experimental plots on the lawn of the Botanical Garden. The seedlings were equally and randomly distributed among four experimental plots corresponding to a full factorial design of competition and drought stress. The competition plots were left with an intact vegetative cover, containing naturally occurring *Lolium perenne, Poa pratensis, Festuca rubra, Ajuga reptans, Bellis perennis, Agrimonia eupatoria*, *Glechoma hederacea, Plantago lanceolata, P. major,* and *Trifolium repens.* The vegetation of the plots without competition was removed by soil tilling and the plots were manually weeded throughout the experiment. The drought plots were covered with a PVC panel roof carried by a 1.5 m high construction, to protect them from rainfall which is particularly abundant in late spring in Silesian Czechia, while allowing for direct sun exposure and air circulation. The plants in this treatment were watered only when they showed pronounced signs of drought stress. The watered plots were left uncovered, thus the plants in this treatment received rainfall and were watered when it was not raining for more than two days in a row. Each experimental plot of dimensions 2.4 × 6.3 m had three rows spaced 60 cm from one another. Within a row there were 20 plants spaced 30 cm apart, which is a typical distance between plants in open natural populations (authors, pers. obs). Seedlings lost in the week following transfer to the common garden were replaced. After the first week, prematurely lost plants (mostly due to herbivory) were not replaced and were excluded from the analyses.

The same experimental protocol was used for the autumn run of the experiment. Seeds were sown on the 23rd of July 2019, seedlings were transplanted in individual planters on the 9th of August, and then up to 28 seedlings per treatment were transferred into the four experimental plots on the 22nd of August (total of 204 plants). The patches without competition were not ploughed in autumn—instead, the soil was covered with plastic foil throughout the summer to prevent the growth of other plants. This resulted in differences in the soil texture between seasons, with the soil of the no competition treatment being visibly more compact in autumn than in spring, and more compact than the soil of natural habitats characterized as early succession. To avoid confounding effects of soil texture and season in the no competition treatment, the data from the two seasons were analysed separately.

### Trait measurement

The plants were checked for the emergence of the first CL and CH flower every other day during the first two weeks after transfer to soil, then every four to five days until the end of the experiment. At the end of the experiment in each season (27th of July in spring and 15th November in autumn), the total number of stems and the total number of calices (i.e. number of flowers) on the principal stem were counted (*N*_ax_ and *N*_cal_, respectively). Both *N*_ax_ and *N*_cal_ are highly and positively correlated with aboveground dry biomass (Stojanova *et al.* 2020).

Chasmogamous flowers on the principal stem were counted every four days, which corresponds to a CH corolla lifetime after blooming. The total number of CH flowers (*N*_CH_) was calculated as the sum of all CH flowers counted during the flowering season. Given that CL flowers cannot be reliably distinguished from young CH buds, the total number of CL flowers (*N*_CL_) was calculated at the end of the flowering season as *N*_CL_ = *N*_cal_—*N*_CH_, and the proportion of CL flowers was calculated as *p*_CL_ = *N*_CL_/*N*_cal_. It has been previously shown that the flower traits measured on the principal stem are highly correlated with the flower traits measured on all stems (Stojanova *et al.* 2020).

Up to five CL and five CH flowers per plant were marked with a dot of acrylic paint on their calix and left to pollinate naturally. To avoid losing seeds, the calyx was closed by gluing its sepals together with a drop of paint after the corolla fell off. Since the number of ovules per flower is invariably four, we estimated the CL and CH fertilization success (G_CH_, G_CL_) as the total number of seeds in the marked flowers, divided by four times the number of marked flowers. Chasmogamous fertilization success was only estimated in the watered treatment as the PVC roof could affect pollinator behaviour. Trait abbreviations are presented in Table 2.

**Table 2.** Trait abbreviations, calculation, and their transformation for subsequent statistical analyses.

Abbreviation	Description	Estimate	Transformation
*d*_CL_, *d*_CH_	Date of first CL and CH flower	Recorded date	None
*G*_CL_, *G*_CH_	CL and CH fertilization success	Seeds in marked flowers /4 × marked flowers	Square root
*N*_ax_	Number of stems	Counted	None
*N*_cal_	Number of flowers (on the main stem)	Counted	None
*N*_CL_	Number of CL flowers	*N*_cal_—*N*_CH_	None
*N*_CH_	Number of CH flowers	Counted	None
p_CL_	Proportion of CL flowers	N_CL_/N_cal_	Square root

### Statistical analyses

All analyses were made in R4.3.2. All traits were analysed with ANOVA with type 3 sums of squares using the glm function from R base package with population, competition, drought and all their interactions as dependent variables. Proportion data (*G*_CL_, *G*_CH_, *p*_CL_) were square-root-transformed to fulfil model requirements of residual distribution and homoscedasticity. The data from the spring and autumn experiment were analysed separately because of the differences in soil texture between seasons that could affect plant development (see above). When a significant effect of population was found, we identified significant differences among populations with post-hoc Tukey tests. When a significant treatment × population interaction was found, for each population we performed separate post-hoc tests to identify significant differences between treatments.

A principal component analysis (PCA) was conducted on standardized variables measured in spring using the package FactoMineR (Lê *et al.* 2008). To avoid problems of missing observations, we averaged trait values across populations and treatments and omitted G_CH_ which was not measured in the drought treatment.

## Results

### Response to competition and drought in spring

**Figure 1.**
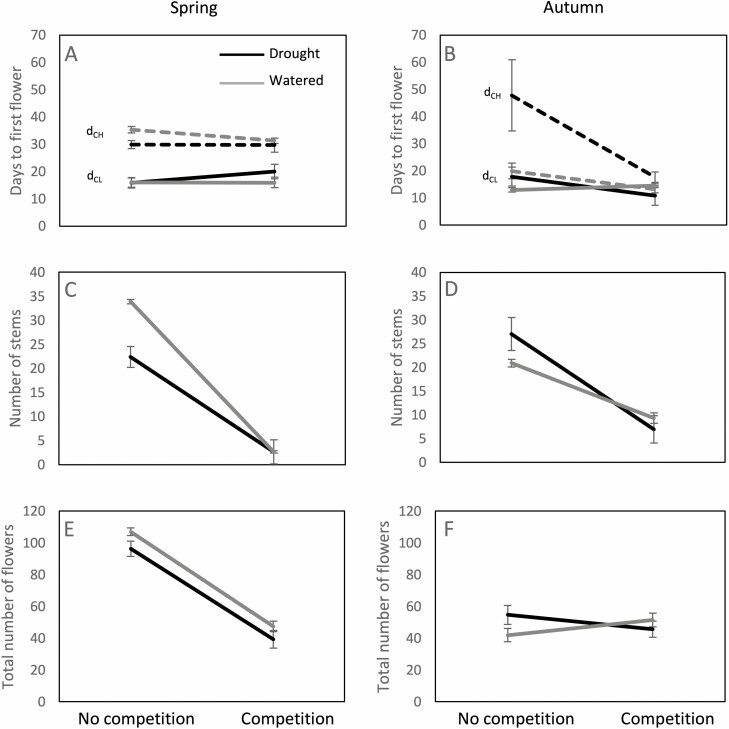
Reaction norms for phenology, vegetative size, and total number of flowers between competition treatments for plants exposed to different watering regimes in spring (left) and autumn (right). A, B—days to the first recorded CH or CL flower in plants that produced at least one CH or CL flower respectively; C, D—number of flowering stems: E, F—total number of flowers on the principal stem. Black lines—drought treatment, grey lines—watered. In A, B full lines correspond to CL flowers, and dashed lines to CH flowers. Error bars correspond to standard errors.

We analysed the start of CL and CH flowering in spring for 98% of the plants that produced at least one CL flower, and for 79% that produced at least one CH flower. On average, CL flowering began about 15–20 days after seedling transplantation in the soil, and CH flowering began 10–15 days after CL flowering. The date of the first CL flower did not vary between treatments. The date of the first CH flower was not affected by competition, but it was significantly advanced by drought by 3.5 days (Table 3, Fig. 1a).

**Table 3.** ANOVA with type 3 sum of squares for phenology, vegetative size, and number of flowers. NULL corresponds to the total degrees of freedom and total variance of the model. Comp—competition treatment. Significant *P*-values are in bold.

	Days to CH			Days to CL			Number of stems			Number of flowers		
Spring	Df	F	Pr(> F)	Df	F	Pr(> F)	Df	F	Pr(> F)	Df	F	Pr(> F)
NULL	166	15 073		215	48 936		216	75 484		218	1477	
Pop	**6**	**13.20**	**<0.001**	**6**	**116.4**	**<0.001**	**6**	**13.51**	**<0.001**	**6**	**33.86**	**<0.001**
Comp	**1**	**4.242**	**0.041**	1	3.697	0.056	**1**	**355.3**	**<0.001**	**1**	**329.9**	**<0.001**
Drought	**1**	**19.86**	**<0.001**	1	0.623	0.431	**1**	**21.57**	**<0.001**	**1**	**5.431**	**0.021**
Pop × comp	6	1.653	0.137	6	1.409	0.213	**6**	**9.456**	**<0.001**	6	1.512	0.176
Pop × drought	5	1.660	0.148	6	0.335	0.918	**6**	**3.529**	**0.002**	6	0.736	0.621
Comp × drought	1	1.163	0.283	1	0.114	0.736	**1**	**18.49**	**<0.001**	1	0.283	0.595
Pop × comp × drought	5	1.582	0.169	6	0.251	0.958	**6**	**2.294**	**0.037**	6	1.668	0.131
Autumn												
NULL	88	15420		167	68285		173	45535		165	1112	
Pop	**6**	**8.085**	**<0.001**	**7**	**126.2**	**<0.001**	7	1.940	0.068	**7**	**50.90**	**<0.001**
Comp	**1**	**20.87**	**<0.001**	1	0.151	0.698	**1**	**58.91**	**<0.001**	1	0.135	0.714
Drought	**1**	**14.45**	**<0.001**	1	0.066	0.798	1	0.282	0.596	1	0.022	0.883
Pop × comp	**5**	**2.897**	**0.020**	7	0.158	0.993	7	0.994	0.438	7	1.163	0.328
Pop × drought	5	0.997	0.427	7	0.394	0.905	**7**	**2.109**	**0.046**	7	0.976	0.452
Comp × drought	**1**	**8.206**	**0.006**	1	0.679	0.411	1	3.033	0.084	**1**	**8.076**	**0.005**
Pop × comp × drought	**3**	**6.882**	**<0.001**	7	0.795	0.593	7	1.357	0.228	7	1.140	0.342

Both competition and drought significantly reduced vegetative size and total number of flowers (Table 3, Fig. 1c and e) and there was a significant competition × drought effect on vegetative size. Specifically, when grown in competition, all plants had only one to three stems regardless of the drought regime, whereas without competition watered plants produced on average 34 stems and plants growing in drought produced 22 stems.

**Figure 2.**
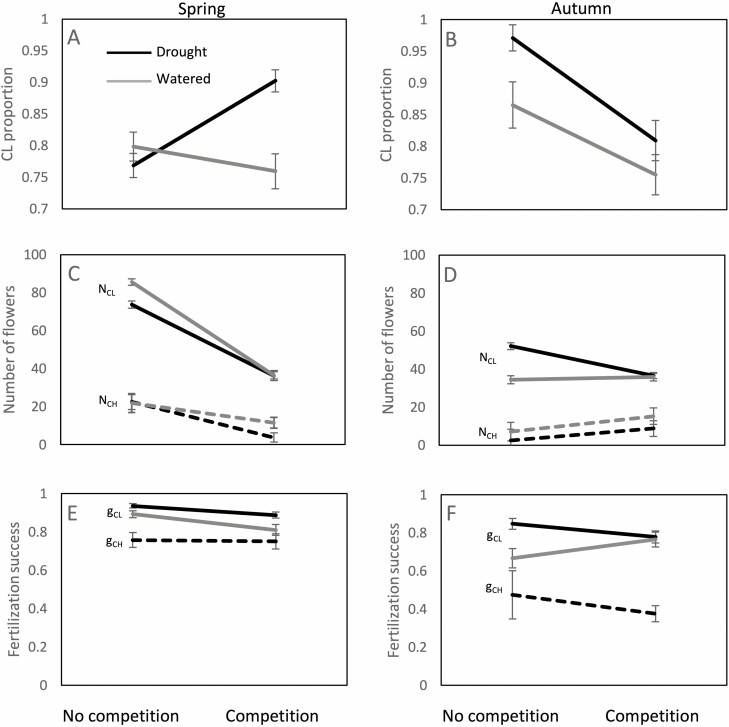
Reaction norms for flower traits and fertilization success between competition treatments for plants exposed to different watering regimes in spring (left) and autumn (right). A, B—CL proportion; C, D—number of CH and CL flowers, E, F—CH and CL fertilization success. Black lines—drought treatment, grey lines—watered. In C-F full lines correspond to CL flowers, dashed lines to CH flowers. Error bars correspond to standard errors.

The competition × drought interaction had significant effect on the CL proportion. CL proportion was significantly higher in the competition-drought treatment (over 90%). In the remaining three treatments no significant differences were observed (CL proportion between 75 and 80%) despite the marked size differences across treatments (Table 4, Fig. 2a). The observed variation in CL proportions across competition and drought treatments was a result of the adjustment of the number of both, CH and CL flowers (Table 4, Fig. 2c). The production of both flower types significantly decreased in response to competition and drought, but the competition × drought interaction had contrasting effects on CH and CL production. When all populations were analysed together, drought significantly decreased CL production in the no competition treatment but not in the competition treatment. Within populations, post-hoc tests did not detect significant effects of the competition × drought interaction, likely because of the low sample size and high variance of individual CL production (see Supporting Information—Fig. S1). The production of CH flowers, on the contrary, was decreased by drought in the competition treatment (Table 4, Fig. 2c). Within populations, post-hoc tests confirmed this pattern for populations CZ1, CZ2, and IL1, and similar qualitative trends were observed in NMK2 and NMK3 (see Supporting Information—Fig. S1).

**Table 4.** ANOVA with type 3 sum of squares for flower traits and fertilization success. NULL corresponds to the total degrees of freedom and total variance of the model. Comp—competition treatment. Significant *P*-values are in bold. CH fertilization success was only measured in the watered treatment hence drought and its interactions were not included in the analysis. In autumn, there were too few observations for CH fertilization success thus no statistical comparisons were made.

	CL proportion			Number of CH flowers			Number of CL flowers			CH fertilization success			CL fertilization success		
Spring	Df	F	Pr(> F)	Df	F	Pr(> F)	Df	F	Pr(> F)	Df	F	Pr(> F)	Df	F	Pr(> F)
NULL	218	12		219	43767		214	268536		89	3.39		204	1	
Pop	**6**	**19.28**	**<0.001**	**6**	**11.05**	**<0.001**	**6**	**23.55**	**<0.001**	5	0.250	0.939	6	0.468	0.832
Comp	**1**	**19.33**	**<0.001**	**1**	**103.9**	**<0.001**	**1**	**211.7**	**<0.001**	1	0.008	0.927	**1**	**12.01**	**0.001**
Drought	**1**	**13.18**	**<0.001**	**1**	**4.418**	**0.037**	1	3.541	0.061				**1**	**11.28**	**0.001**
Pop × comp	**6**	**2.521**	**0.023**	**6**	**3.128**	**0.006**	**6**	**3.786**	**0.001**	5	0.737	0.598	**6**	**3.235**	**0.005**
Pop × drought	6	1.313	0.253	6	0.854	0.530	6	0.542	0.776				6	1.656	0.134
Comp × drought	**1**	**26.29**	****<**0.001**	**1**	**6.985**	**0.009**	**1**	**6.179**	**0.014**				1	0.862	0.354
Pop × comp × drought	6	0.820	0.555	6	0.978	0.442	6	0.784	0.584				5	0.844	0.520
Autumn															
NULL	165	14		170	28882		165	121866			NA		141	5	
Pop	**7**	**11.13**	**<0.001**	**7**	**6.334**	**<0.001**	**7**	**30.40**	**<0.001**				**7**	**4.598**	**<0.001**
Comp	**1**	**44.61**	**<0.001**	**1**	**18.61**	**<0.001**	**1**	**9.333**	**0.003**				1	0.173	0.678
Drought	**1**	**19.58**	**<0.001**	**1**	**13.47**	**<0.001**	**1**	**5.578**	**0.020**				**1**	**8.746**	**0.004**
Pop × comp	**7**	**2.146**	**0.043**	7	1.009	0.428	7	1.563	0.152				**7**	**3.771**	**0.001**
Pop × drought	7	1.243	0.284	7	0.577	0.773	7	1.111	0.360				**6**	**3.253**	**0.005**
Comp × drought	1	2.799	0.097	1	0.542	0.463	**1**	**9.515**	**0.002**				**1**	**5.722**	**0.018**
Pop × comp × drought	7	1.392	0.214	7	0.917	0.496	7	0.715	0.660				**5**	**3.245**	**0.009**

Cleistogamous fertilization success was very high (above 80%) in all treatments. Competition and drought, but not their interaction, significantly decreased CL fertilization success (Table 4, Fig. 2e). This is a somewhat surprising result, as CL flowers are considered to have reliable pollination whose success does not vary in response to environmental conditions. Fertilization success of CH flowers was about 75% and did not vary between the competition and no competition treatments (Table 4, Fig. 2e).

### Response to competition and drought in autumn

On average, plants in autumn had reduced vegetative size, fewer flowers than in spring, higher proportions of CL flowers in the absence of competition, and lower CL and CH fertilization success (Figs. 1d and f; 2b and f).

Ninety-five percent of the plants produced CL flowers in autumn, whereas the percentage of plants producing CH flowers was lower in autumn than in spring—only 51% produced at least one CH flower, going as low as 10% in the no competition-drought treatment. The pattern of CL flowering did not differ in autumn compared to spring and was not affected by any of the treatments (Table 3, Fig. 1b). CH flowering was advanced in comparison to spring, meaning that the few plants that produced CH flowers in autumn did so as early as possible. An exception to this was the control-drought treatment, where the few plants that produced CH flowers did so considerably later (Table 3, Fig 1b). Contrary to spring, drought increased vegetative growth and flower production, especially in the absence of competition (Table 3, Fig. 1d, f). Competition significantly decreased, while drought significantly increased the CL proportion, whereas the competition × drought interaction had no significant effect (Table 4, Fig. 2b). The number of CL flowers was affected by competition, drought, and their interaction. Plants without competition produced more CL flowers when watered than when exposed to drought. Plants with competition produced the same number of CL flowers regardless of the drought regime (Table 4, Fig. 2d). The number of CH flowers significantly increased in response to competition and drought (Table 4, Fig 2d). Post-hoc tests did not detect any consistent patterns, likely because of the overall smaller sample size per population and per treatment in autumn (see Supporting Information—Fig. S2). Finally, CH fertilization success decreased in autumn compared to spring (Fig. 2f), but comparisons between treatments in autumn were not possible because of the small sample sizes.

### Population differentiation

**Figure 3.**
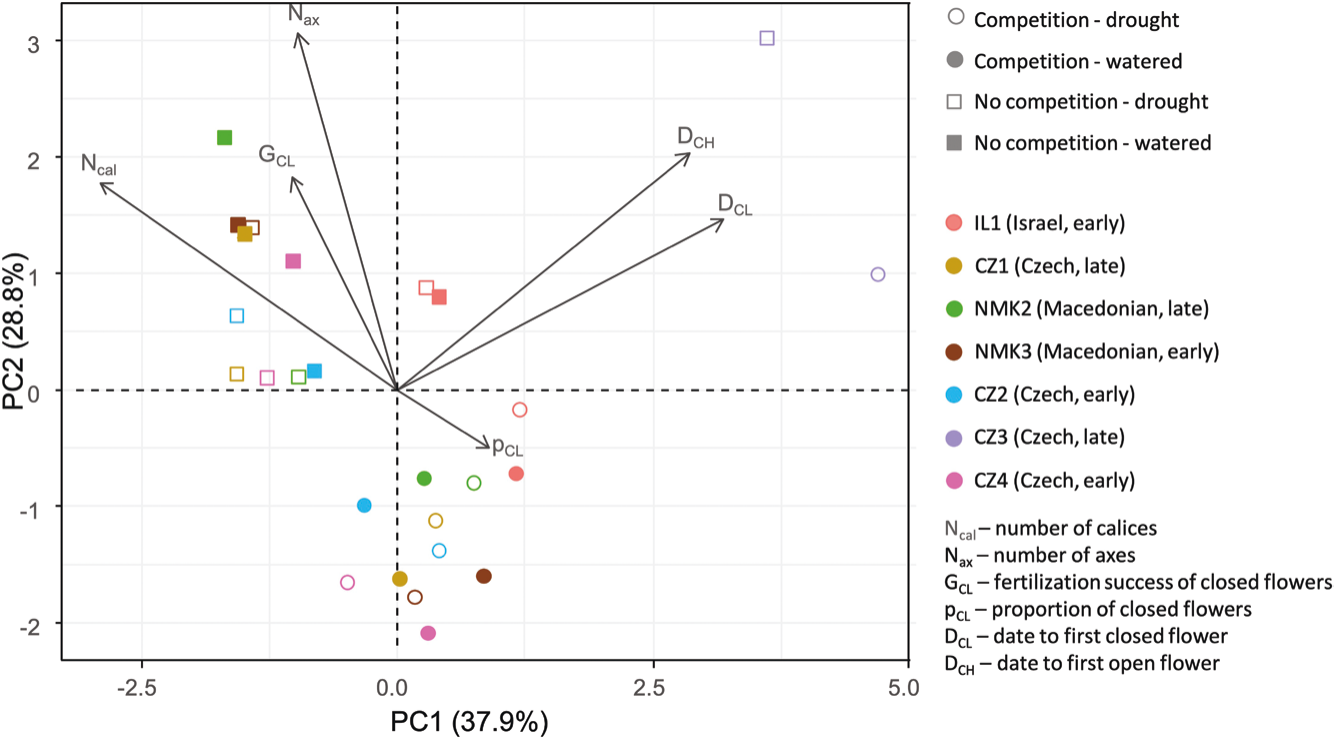
PCA biplot of the first two principal components for traits measured in spring. Each symbol corresponds to the population mean measured in a specific experimental treatment. Arrows indicate the variable loadings.

Population had a significant effect on most traits in both seasons, except for CH and CL fertilization success in spring, suggesting population differentiation for the means of most studied traits (Tables 3 and 4, see Supporting Information—Fig. S3). We also observed population × competition or population × drought interactions for number of stems, CL proportion, and CL fertilization success in spring, and for number of stems and CL flowering date in autumn, suggesting population differentiation for phenotypic plasticity of these traits (Tables 3 and 4, see Supporting Information—Fig. S3). Principal component analyses showed that the first PC axis was highly correlated (correlation coefficient > 0.8) with the start of CL and CH flowering and with number of flowers (Fig. 3). The second axis was highly correlated with number of stems (Fig. 3, and the third with CL proportion (not shown).

According to the PCA, the populations were not grouped by habitat type nor climate (Fig. 3, see Supporting Information—Fig. S3). Instead, five of the six European populations grouped together and showed similar phenotypes, and were differentiated from populations CZ3 (Czech, late succession) and IL1 (Israel). The cluster of five European populations was maintained even when CZ3 and IL1 were removed from the analysis (not shown). Regarding the outlier populations, CZ3 plants were much smaller, flowered later than any other population, and had higher CL proportions (see Supporting Information—Fig. S3). The IL1 population had a remarkably lower CL proportion than any other population, with smaller individuals than the European populations (see Supporting Information—Fig. S3).

## Discussion

### In spring, competition and drought affect cleistogamy and fitness in a non-additive manner

Competition and drought both have negative effects on fitness in spring, suggesting these are unfavourable conditions for *Lamium amplexicaule*. This result is in line with previous detailed observations of French populations of *L. amplexicaule* (Stojanova *et al.* 2014) and with general trends of plant growth in the sampled Czech and Macedonian populations (authors, pers. obs.). In all three countries plants are smaller in densely colonized, late-succession habitats (prairies, lawns, field margins) compared to recently disturbed, uncolonized, early succession habitats (ploughed fields and vineyards). These results are not surprising, as the species is characterized as ruderal (Grime 1977), meaning that it performs best in early succession environments with few resource limitations. The effects of competition and drought were not strictly additive, as all plants exposed to competition are comparable in size, usually producing only one flowering stem, regardless of the drought treatment.

Competition and drought decreased both, CH and CL flower production, but their combined effect was non-additive and acted in opposing directions for CH and CL flowers. Thus, CL proportion and plant size were not related across treatments—plants exposed to competition were always very small and produced fewer flowers, yet only plants exposed to competition and drought at the same time increased their CL proportion. In the remaining three treatments, plants have comparable CL proportions, despite significant differences in size. In line with this, the number of stems did not have a significant effect on CL proportion (not shown). This pattern can be due to the regulation of the production of CL and CH flowers in *L. amplexicaule*—the species starts flowering by producing exclusively CL flowers (constitutive cleistogamy, Lord 1979), and then switches to producing CH and CL flowers in variable proportions in response to environmental cues indicating suitable conditions for CH flowering (plastic cleistogamy), regardless of how the environment affects plant growth. In line with this, Falik *et al.* (2024) observed a decrease of the CL proportion in individuals grown at high intraspecific densities and in individuals exposed to root leachates obtained from other *L. amplexicaule* plants grown at high densities. Our study additionally shows that when exposed to extreme environments combining multiple stressors, the plastic stage of cleistogamy might not be reached, because of additional physiological limitations on plant growth.

### Effects of drought and competition differ between seasons

Competition and drought combined seem to have inverse effects on plant size in autumn compared to spring (number of stems and flowers), with plants growing larger when exposed to drought only, but not to drought and competition simultaneously. Previous studies of the effect of seasonal variation on *L. amplexicaule* grown in pots have also yielded inconsistent results, with some populations growing larger plants in autumn, and others in spring (Stojanova *et al.* 2016, 2020). However, it is possible that the differences between no-competition treatments across seasons in this study, in particular the more compact soil in autumn, could have biased the results. Compact soils generally restrict root growth, nutrient and water uptake, and even in the absence of nutrient limitation can trigger hormonal signals to slow down aboveground growth (Passioura 1991). It is thus plausible that without the differences in soil texture, the response to competition and drought would not differ between seasons.

The CL proportion was overall higher in autumn than in spring, in line with previous studies of seasonal variation of cleistogamy in *L. amplexciaule* (Stojanova *et al.* 2016, 2020). In spring notably, competition and drought independently increased the relative production of CL flowers, but their interaction was not significant. Summer annuals of *L. amplexicaule* (i.e. plants germinating in late summer and flowering in autumn) have been only documented in early succession habitats, presumably because reduced precipitation, dry weather, and intraspecific competition impede seed germination in summer (Baskin and Baskin 1981). It is thus possible that the observed patterns of cleistogamy variation in autumn are non-adaptive since natural populations of *L. amplexicaule* do not experience competitive environments in autumn.

### Identification of three ecotypes which do not differentiate by habitat type

Multivariate analyses did not group populations neither by climate nor by habitat type. The lack of differentiation between early- and late-succession-habitat populations could be due to the fact that the late succession populations were not adapted to the habitat in which they were sampled. Indeed, an inspection of the sites of populations NMK1, NMK2 and CZ1 in 2020 and 2021 did not find any *L. amplexicaule* plants, suggesting that these populations have been the result of fortuitous germination of a few accidentally dispersed seeds from a nearby open population.

Two populations growing in habitats with the most extreme climate and photoperiod did not group with the remaining five according to the PCA. Population CZ3 (late succession habitat, temperate climate) showed significantly delayed flowering patterns, and had the largest vegetative size, both of which are typical for a winter ecotype growing out of season (Stojanova *et al.* 2016). Winter annuals germinate in autumn and overwinter in a vegetative stage, then start flowering at the same time as spring annuals (Baskin and Baskin 1981). When germinated in spring, winter annuals tend to have a very long and prolific vegetative stage followed by delayed flowering and only a few flowers (Stojanova *et al.* 2016).

Population IL1 (early succession habitat, desert climate) had a remarkably lower CL proportion compared to any other population, the least variation in CL proportion across experimental treatments, and multiple plants that started flowering by producing CH flowers first. It is possible that this population has adapted to flowering by warm weather with short photoperiods since at harvest time, photoperiod in the natural populations was 10–11 h daylight whereas in European populations it was 12–14 h. It is well established that photoperiod and temperature are two factors that highly influence the timing of flowering in non-cleistogamous (Amasino 2010) and variation in cleistogamous species (Lord 1982; Oakley *et al.* 2007). Whether the change in flowering patterns in the Israeli population is due to genetic shifts in response to an atypical combination of environmental cues for *L. amplexicaule*, needs to be tested in an environment with controlled photoperiod and temperature.

### Is variation in cleistogamy an adaptive strategy? Future research directions

The increase of CL proportion in the competition-drought treatment relative to all other treatments could be a means for *L. amplexicaule* to maintain a certain reproductive output even when resources highly limit plant growth and flower production, in line with the resource adaptation hypothesis as proposed by Schemske (1978). However, the competition-watered treatment reduces plant growth and total flower production in a similar manner, without decreasing the CL proportion. It is thus also plausible that as long as environmental conditions allow physiological switching to the production of CH flowers, *L. amplexicaule* will invest in the production of these costly flowers because of other adaptive advantages they may confer. Below we discuss possible explanations of the observed patterns of CL proportions, and how to further test these hypotheses using *L. amplexicaule*.

It has been suggested that cleistogamous species are able to predict pollen limitation in their environment and decrease CH production to provide reproductive assurance without entailing undesired costs such as those caused by prior or facilitated selfing in non-cleistogamous species (Lord 1982; Morgan and Wilson 2005). In line with this, field observations have shown higher CH fertilization success in *L. amplexicaule* populations growing in late-succession habitats (Stojanova *et al.* 2014), which bear more diverse flora and could thus sustain more abundant and diverse pollinator communities. However, differences in CH fertilization success between treatments were only marginal in this common garden study, thus precluding any reliable inference about the role of pollination environment. A likely explanation is that the pollinator activity between treatments did not differ between adjacent plots in the common garden. Moreover, CL fertilization success was significantly higher than CH fertilization success across all treatments in this, and other studies of *L. amplexicaule* (Stojanova *et al.* 2016, 2020). Winn and Moriuchi (2009) similarly found higher CL fertilization success after manipulating CH and CL production of *Viola septemloba* in different environments, suggesting that the mere reproductive output of CH flowers cannot compensate for the reliability and reduced cost of reproduction through CL flowers. Estimating CH and CL fertilization success in natural pollination environments is required to definitively confirm or rule out the effect of pollinators on the reproductive output of the two floral moprhs in cleistogamous species. In addition to this, we suggest accounting for qualitative differences in CL and CH offspring (see below).

Inbred offspring of *L. amplexicaule* do not suffer from inbreeding depression (Stojanova *et al.* 2020). This is likely because of the overall high selfing rates in the species which effectively purge deleterious alleles (Stojanova *et al* 2014). More generally, inbreeding depression is low in cleistogamous species (Trapp and Hendrix 1988; Culley 2000; Munguía-Rosas *et al.* 2013; Ansaldi *et al.* 2019). It is thus unlikely that adjusting the CL proportion is a way to reduce deleterious effects of inbreeding in highly competitive environments as it has been observed for non-cleistogamous species (Cheptou *et al.* 2000). Beyond the effects of inbreeding, Stojanova *et al* (2020) showed that the fitness of CL and selfed-CH offspring varied across seasons, with autumn increasing the fitness of CL, and spring increasing the fitness CH offspring. Thus reproducing through CH and CL flowers could confer non-genetic, environment-dependent offspring advantage in environments that stimulate their production. Assessing the effect of interspecific competition on CL and CH offspring fitness while accounting for inbreeding in CH offspring is required to provide evidence about the adaptive role of the two floral types in response to competition and drought.

## Conclusion

The variation of cleistogamy has been studied across a vast range of environmental cues, including intraspecific competition. Here, we contribute to this knowledge by explicitly testing the effect of interspecific competition, drought, and seasonal variation on the degree of cleistogamy in popululations of an annual ruderal sampled across constrasting environments. The CL proportion tends to decrease in response to competition, despite the negative effects of this treatment on plant growth and total flower number. Only when plants are exposed to drought and competition simultaneously, does the CL proportion increase. We propose to further test if the observed variation could be an adjustment to variation in pollinator abundance and diversity between habitats in different succession stages, as well as an adjustment of the mating system to produce more offspring resilient to competition. Since *Lamium amplexicaule* offspring do not seem to suffer from inbreeding depression (Stojanova *et al.* 2020), it is likely that other, non-genetic factors could favour the production of CH flowers in competitive, late-succession environments. Explicitly testing whether each floral type has a fitness advantage in the environment that enhances its production would be the next step required to demonstrate the adaptive plasticity of cleistogamy in response to intraspecific competition.

## Supporting Information

The following additional information is available in the online version of this article –

Figure S1—Barplots of the total number of CH (in blue) and CL flowers (in orange) per treatment for each of the studied populations in spring. Bars marked with different letters have statistically different means according to Tukey post-hoc tests. Comparisons were made for each flower type separately (corresponding to letters with different colours). The names of the populations are in Table 1 in the main text, late and early refer to the habitat succession in their site of origin. Error bars correspond to standard errors.

Figure S2—Barplots of the total number of CH (in blue) and CL flowers (in orange) per treatment for each of the studied populations in autumn. Bars marked with different letters have statistically different means according to Tukey post-hoc tests. Comparisons were made for each flower type separately (corresponding to letters with different colours). The names of the populations are in Table 1 in the main text, late and early refer to the habitat succession in their site of origin. Error bars correspond to standard errors.

Figure S3—Barplots of trait means per population and treatments in spring. Full bars—competition, hatched bars—no competition, black—drought, grey—watered treatment. Populations with statistically different means according to Tukey post-hoc test are marked with different letters above the horizontal line on top of the bars. The names of the populations are in Table 1 in the main text, late and early refer to the habitat succession in their site of origin. Error bars correspond to standard errors.

plae036_suppl_Supplementary_Materials

## Data Availability

The data underlying this article are available in Zenodo repository, at https://zenodo.org/doi/10.5281/zenodo.11526946
