# Supplementary material for: Competition and drought affect cleistogamy in a non-additive way in the annual ruderal Lamium amplexicaule
Source: AoB Plants. 2024 Jun 24;16(4):plae036. doi: 10.1093/aobpla/plae036 (PMC11232460; doi:10.1093/aobpla/plae036)
Supplement: plae036_suppl_Supplementary_Materials [file plae036_suppl_supplementary_materials.pptx]

## Slide 1
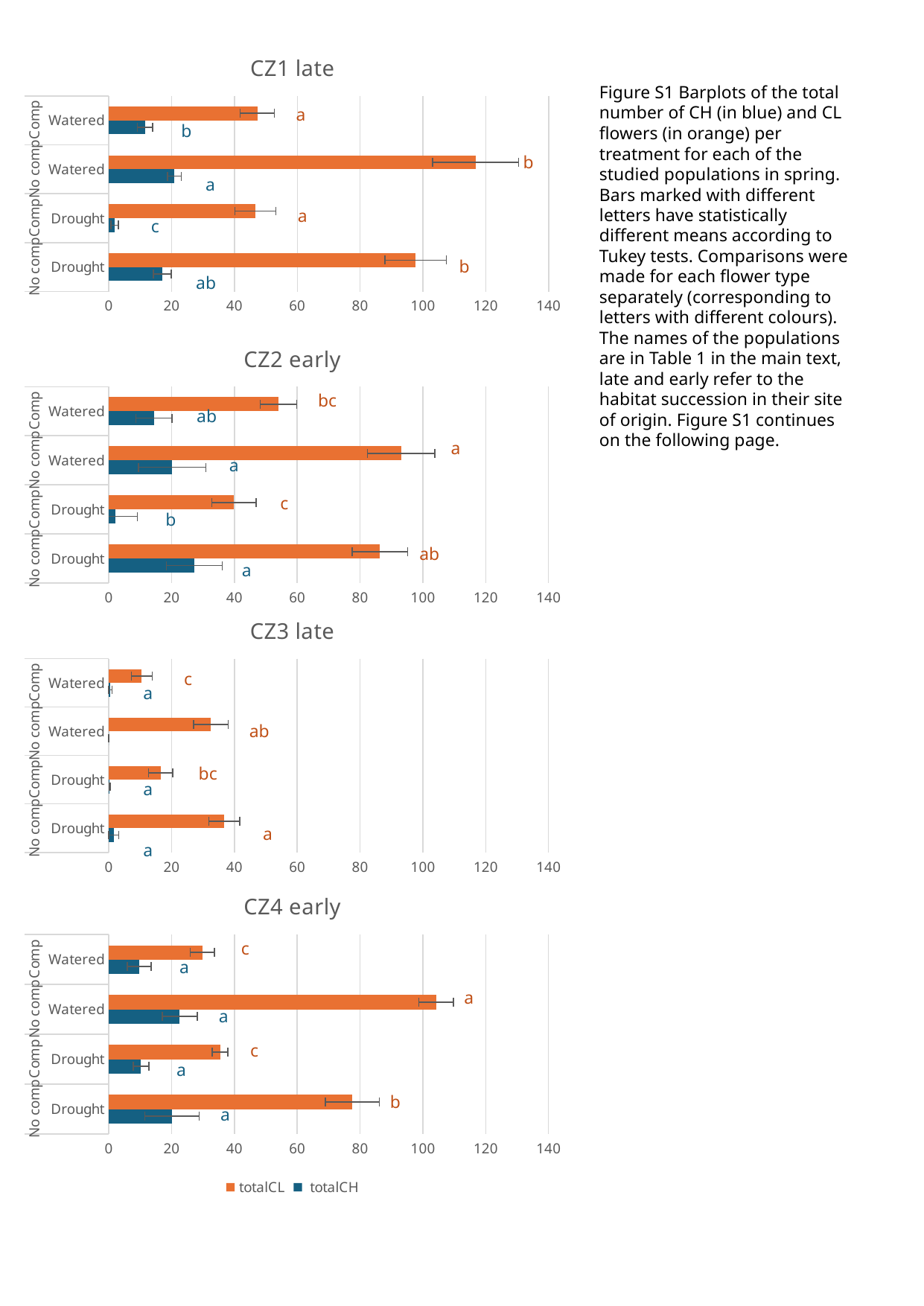

### Chart: CZ1 late
| Category | totalCH | totalCL |
|---|---|---|
| Drought | 17.0 | 97.66666667 |
| Drought | 1.888888889 | 46.66666667 |
| Watered | 20.88888889 | 116.7777778 |
| Watered | 11.5 | 47.25 |a
b
b
a
a
c
b
ab
Figure S1 Barplots of the total number of CH (in blue) and CL flowers (in orange) per treatment for each of the studied populations in spring. Bars marked with different letters have statistically different means according to Tukey tests. Comparisons were made for each flower type separately (corresponding to letters with different colours). The names of the populations are in Table 1 in the main text, late and early refer to the habitat succession in their site of origin. Figure S1 continues on the following page.
### Chart: CZ2 early
| Category | totalCH | totalCL |
|---|---|---|
| Drought | 27.22222222 | 86.33333333 |
| Drought | 2.0 | 39.875 |
| Watered | 20.2 | 93.11111111 |
| Watered | 14.375 | 54.0 |bc
ab
a
a
c
b
ab
a
### Chart: CZ3 late
| Category | totalCH | totalCL |
|---|---|---|
| Drought | 1.6 | 36.8 |
| Drought | 0.25 | 16.5 |
| Watered | 0.0 | 32.5 |
| Watered | 0.5 | 10.5 |c
a
ab
bc
a
a
a
### Chart: CZ4 early
| Category | totalCH | totalCL |
|---|---|---|
| Drought | 20.11111111 | 77.55555556 |
| Drought | 10.25 | 35.42857143 |
| Watered | 22.55555556 | 104.2222222 |
| Watered | 9.666666667 | 29.77777778 |c
a
a
a
c
a
b
a

## Slide 2
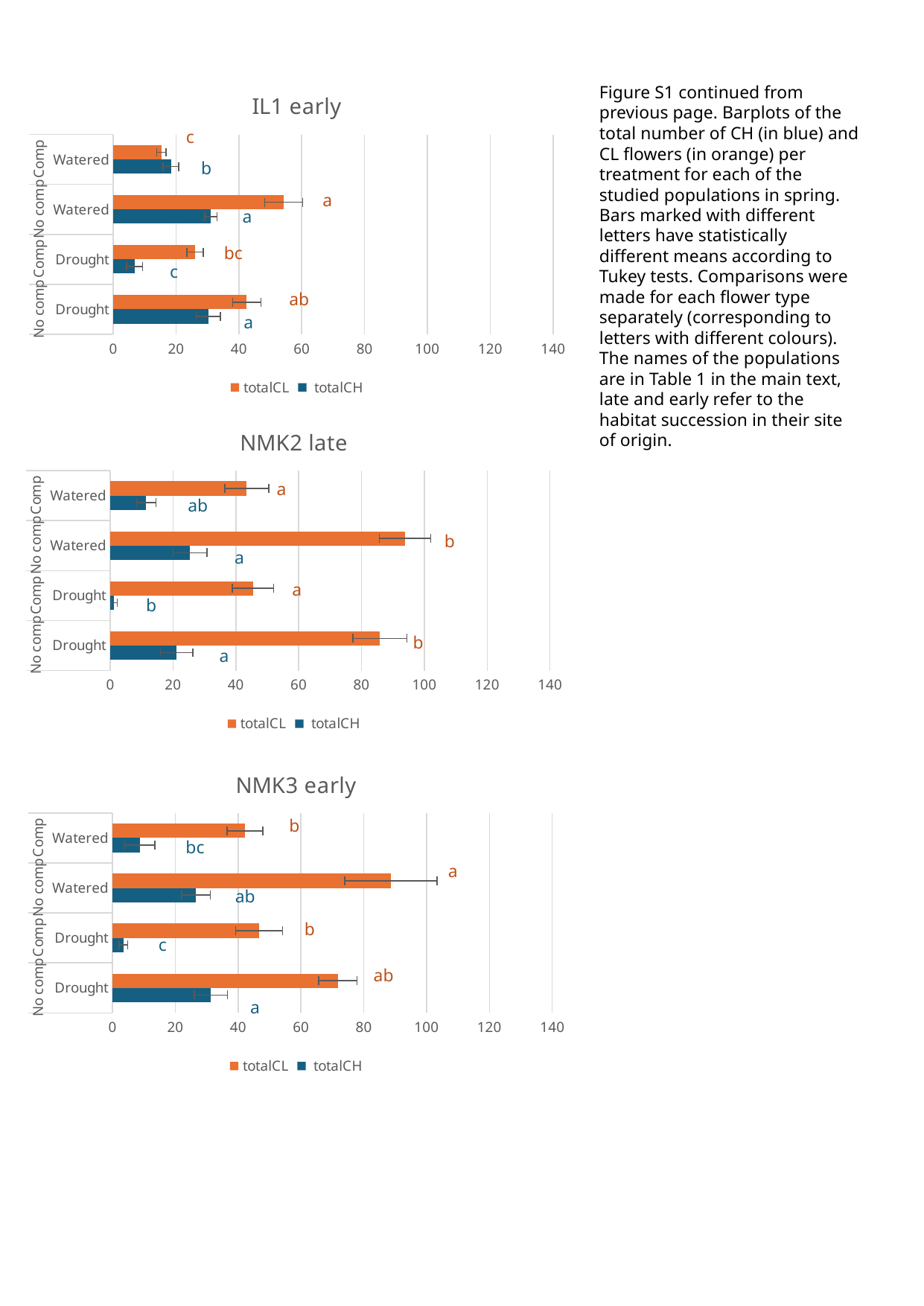

### Chart: IL1 early
| Category | totalCH | totalCL |
|---|---|---|
| Drought | 30.25 | 42.5 |
| Drought | 6.777777778 | 26.0 |
| Watered | 31.11111111 | 54.22222222 |
| Watered | 18.375 | 15.28571429 |c
b
a
a
bc
c
ab
a
Figure S1 continued from previous page. Barplots of the total number of CH (in blue) and CL flowers (in orange) per treatment for each of the studied populations in spring. Bars marked with different letters have statistically different means according to Tukey tests. Comparisons were made for each flower type separately (corresponding to letters with different colours). The names of the populations are in Table 1 in the main text, late and early refer to the habitat succession in their site of origin.
### Chart: NMK2 late
| Category | totalCH | totalCL |
|---|---|---|
| Drought | 21.14285714 | 85.85714286 |
| Drought | 1.285714286 | 45.42857143 |
| Watered | 25.42857143 | 93.85714286 |
| Watered | 11.42857143 | 43.42857143 |a
ab
b
a
a
b
b
a
### Chart: NMK3 early
| Category | totalCH | totalCL |
|---|---|---|
| Drought | 31.33333333 | 71.77777778 |
| Drought | 3.5 | 46.66666667 |
| Watered | 26.55555556 | 88.625 |
| Watered | 8.666666667 | 42.16666667 |b
bc
a
ab
b
c
ab
a

## Slide 3
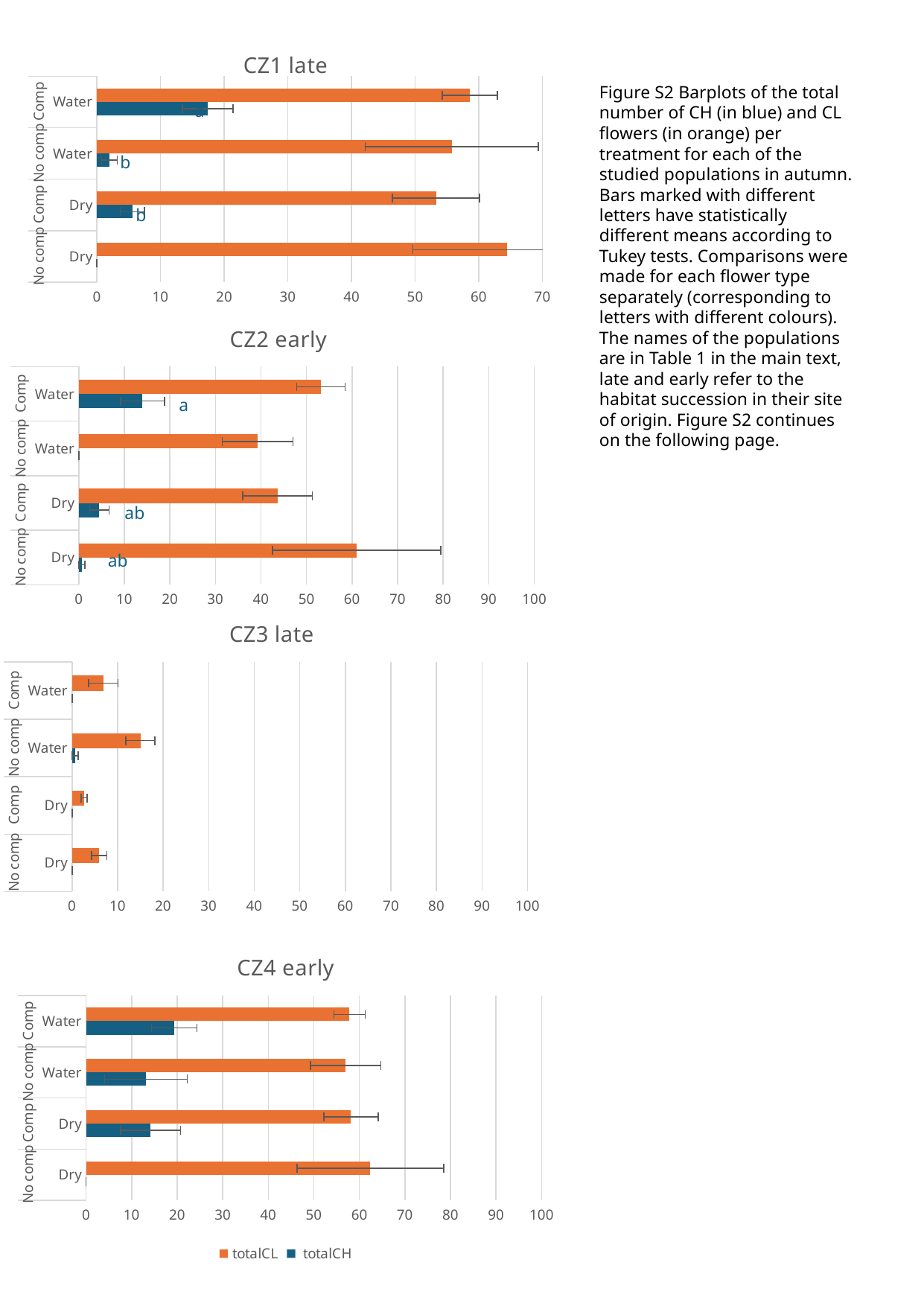

### Chart: CZ1 late
| Category | totalCH | totalCL |
|---|---|---|
| Dry | 0.0 | 64.4 |
| Dry | 5.57142857142857 | 53.2857142857143 |
| Water | 2.0 | 55.75 |
| Water | 17.4285714285714 | 58.5714285714286 |a
b
b
Figure S2 Barplots of the total number of CH (in blue) and CL flowers (in orange) per treatment for each of the studied populations in autumn. Bars marked with different letters have statistically different means according to Tukey tests. Comparisons were made for each flower type separately (corresponding to letters with different colours). The names of the populations are in Table 1 in the main text, late and early refer to the habitat succession in their site of origin. Figure S2 continues on the following page.
### Chart: CZ2 early
| Category | totalCH | totalCL |
|---|---|---|
| Dry | 0.666666666666667 | 61.0 |
| Dry | 4.5 | 43.6666666666667 |
| Water | 0.0 | 39.25 |
| Water | 14.0 | 53.1666666666667 |a
ab
ab
### Chart: CZ3 late
| Category | totalCH | totalCL |
|---|---|---|
| Dry | 0.0 | 6.0 |
| Dry | 0.0 | 2.66666666666667 |
| Water | 0.666666666666667 | 15.0 |
| Water | 0.0 | 6.83333333333333 |
### Chart: CZ4 early
| Category | totalCH | totalCL |
|---|---|---|
| Dry | 0.0 | 62.4 |
| Dry | 14.1666666666667 | 58.1666666666667 |
| Water | 13.1666666666667 | 57.0 |
| Water | 19.3333333333333 | 57.8333333333333 |

## Slide 4
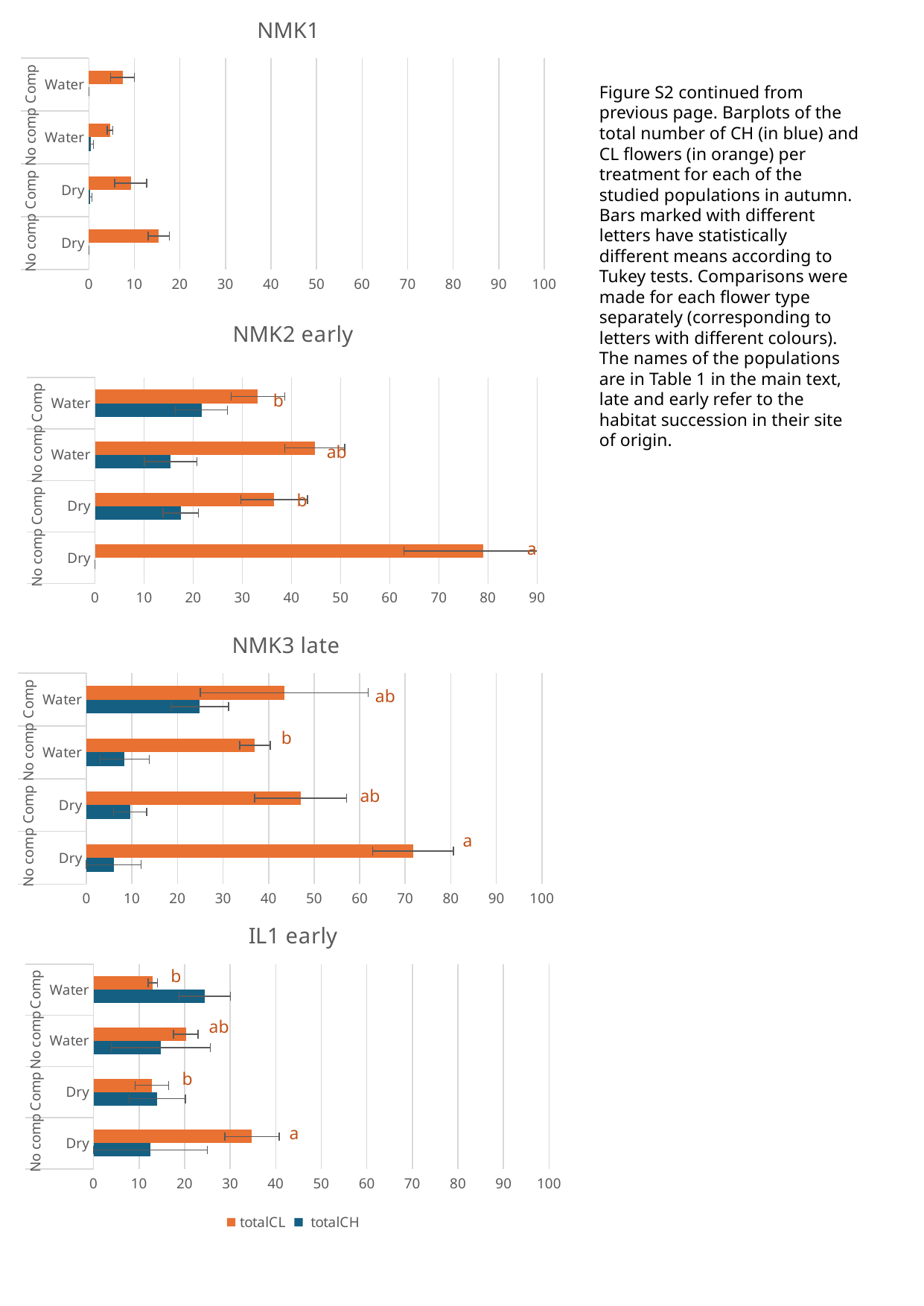

### Chart: NMK1
| Category | totalCH | totalCL |
|---|---|---|
| Dry | 0.0 | 15.3333333333333 |
| Dry | 0.333333333333333 | 9.2 |
| Water | 0.5 | 4.66666666666667 |
| Water | 0.0 | 7.4 |Figure S2 continued from previous page. Barplots of the total number of CH (in blue) and CL flowers (in orange) per treatment for each of the studied populations in autumn. Bars marked with different letters have statistically different means according to Tukey tests. Comparisons were made for each flower type separately (corresponding to letters with different colours). The names of the populations are in Table 1 in the main text, late and early refer to the habitat succession in their site of origin.
### Chart: NMK2 early
| Category | totalCH | totalCL |
|---|---|---|
| Dry | 0.0 | 79.0 |
| Dry | 17.4285714285714 | 36.5 |
| Water | 15.4 | 44.75 |
| Water | 21.6666666666667 | 33.1666666666667 |b
ab
b
a
### Chart: NMK3 late
| Category | totalCH | totalCL |
|---|---|---|
| Dry | 6.0 | 71.7142857142857 |
| Dry | 9.57142857142857 | 47.0 |
| Water | 8.4 | 37.0 |
| Water | 24.8571428571429 | 43.4285714285714 |ab
b
ab
a
### Chart: IL1 early
| Category | totalCH | totalCL |
|---|---|---|
| Dry | 12.5 | 34.75 |
| Dry | 14.0 | 12.8 |
| Water | 14.75 | 20.25 |
| Water | 24.4 | 13.0 |b
ab
b
a

## Slide 5
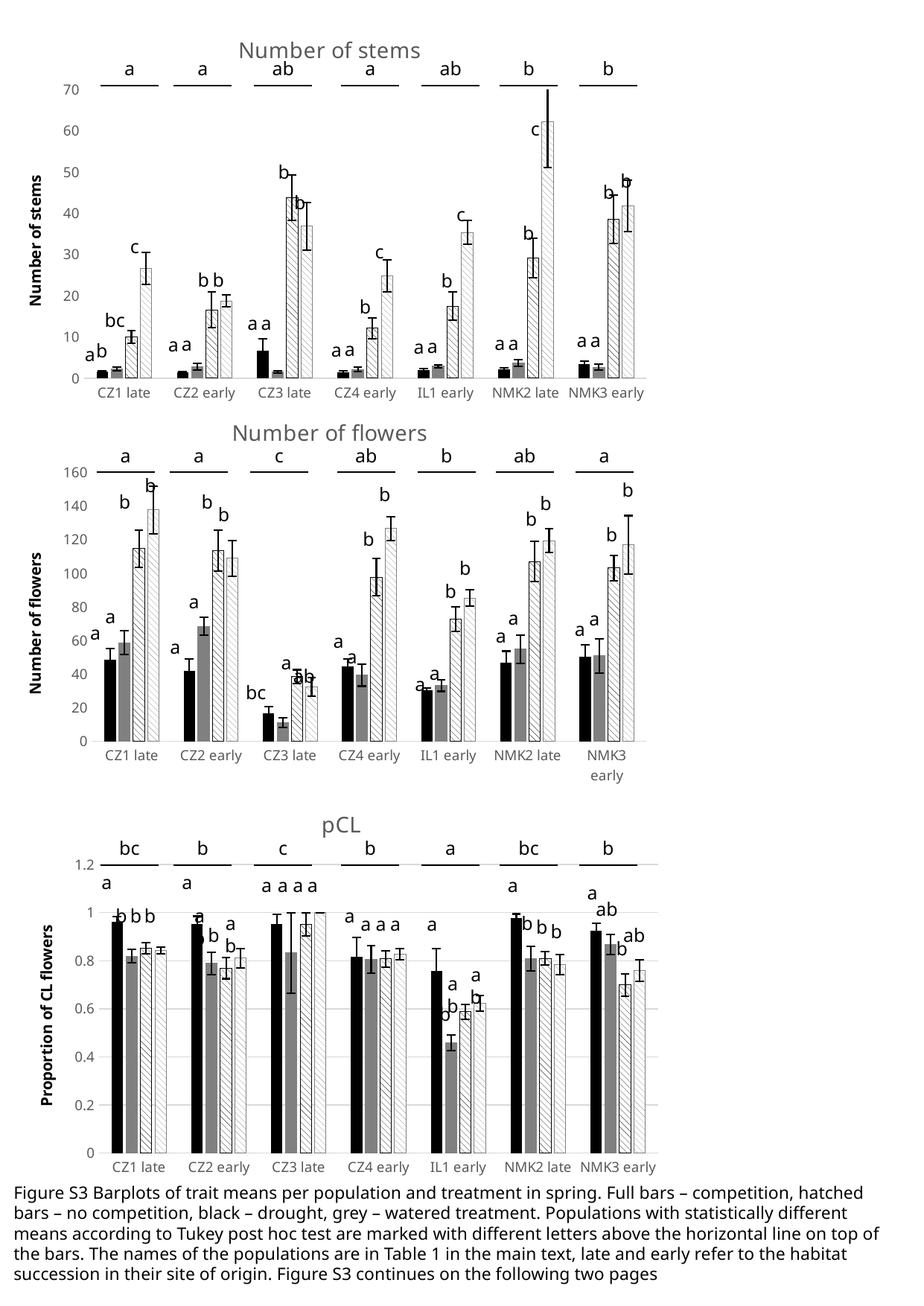

### Chart: Number of stems
| Category | axes | axes | axes | axes |
|---|---|---|---|---|
| CZ1 late | 1.555555556 | 2.285714286 | 10.0 | 26.66666667 |
| CZ2 early | 1.375 | 2.75 | 16.55555556 | 18.7 |
| CZ3 late | 6.75 | 1.5 | 43.75 | 36.83333333 |
| CZ4 early | 1.5 | 2.125 | 12.11111111 | 24.88888889 |
| IL1 early | 2.0 | 2.875 | 17.5 | 35.33333333 |
| NMK2 late | 2.142857143 | 3.714285714 | 29.14285714 | 62.14285714 |
| NMK3 early | 3.333333333 | 2.666666667 | 38.55555556 | 41.77777778 |a
ab
a
ab
b
b
a
c
b
b
b
b
c
b
c
c
b
b
b
b
bc
a
a
a
a
a
a
a
a
a
a
a
a
b
a
### Chart: Number of flowers
| Category | axes | axes | axes | axes |
|---|---|---|---|---|
| CZ1 late | 48.55555556 | 58.75 | 114.6666667 | 137.6666667 |
| CZ2 early | 41.875 | 68.375 | 113.5555556 | 109.0 |
| CZ3 late | 16.625 | 11.0 | 38.4 | 32.5 |
| CZ4 early | 44.5 | 39.44444444 | 97.66666667 | 126.7777778 |
| IL1 early | 30.33333333 | 33.125 | 72.75 | 85.33333333 |
| NMK2 late | 46.71428571 | 54.85714286 | 107.0 | 119.2857143 |
| NMK3 early | 50.16666667 | 50.83333333 | 103.1111111 | 117.0 |ab
ab
a
c
b
a
a
b
b
b
b
b
b
b
b
b
b
b
b
a
a
a
a
a
a
a
a
a
a
a
a
ab
a
bc
c
### Chart: pCL
| Category | axes | axes | axes | axes |
|---|---|---|---|---|
| CZ1 late | 0.961111111 | 0.81875 | 0.852222222 | 0.843333333 |
| CZ2 early | 0.95125 | 0.7887500000000001 | 0.768888889 | 0.8109999999999999 |
| CZ3 late | 0.95125 | 0.8325 | 0.952 | 1.0 |
| CZ4 early | 0.8175 | 0.8055555560000001 | 0.807777778 | 0.826666667 |
| IL1 early | 0.757777778 | 0.45875 | 0.5875 | 0.622222222 |
| NMK2 late | 0.978571429 | 0.808571429 | 0.81 | 0.784285714 |
| NMK3 early | 0.925 | 0.868333333 | 0.698888889 | 0.75875 |b
c
b
a
bc
b
bc
a
a
a
a
a
a
a
a
ab
b
ab
a
b
b
ab
b
a
a
a
a
b
b
b
ab
b
ab
ab
b
Figure S3 Barplots of trait means per population and treatment in spring. Full bars – competition, hatched bars – no competition, black – drought, grey – watered treatment. Populations with statistically different means according to Tukey post hoc test are marked with different letters above the horizontal line on top of the bars. The names of the populations are in Table 1 in the main text, late and early refer to the habitat succession in their site of origin. Figure S3 continues on the following two pages

## Slide 6
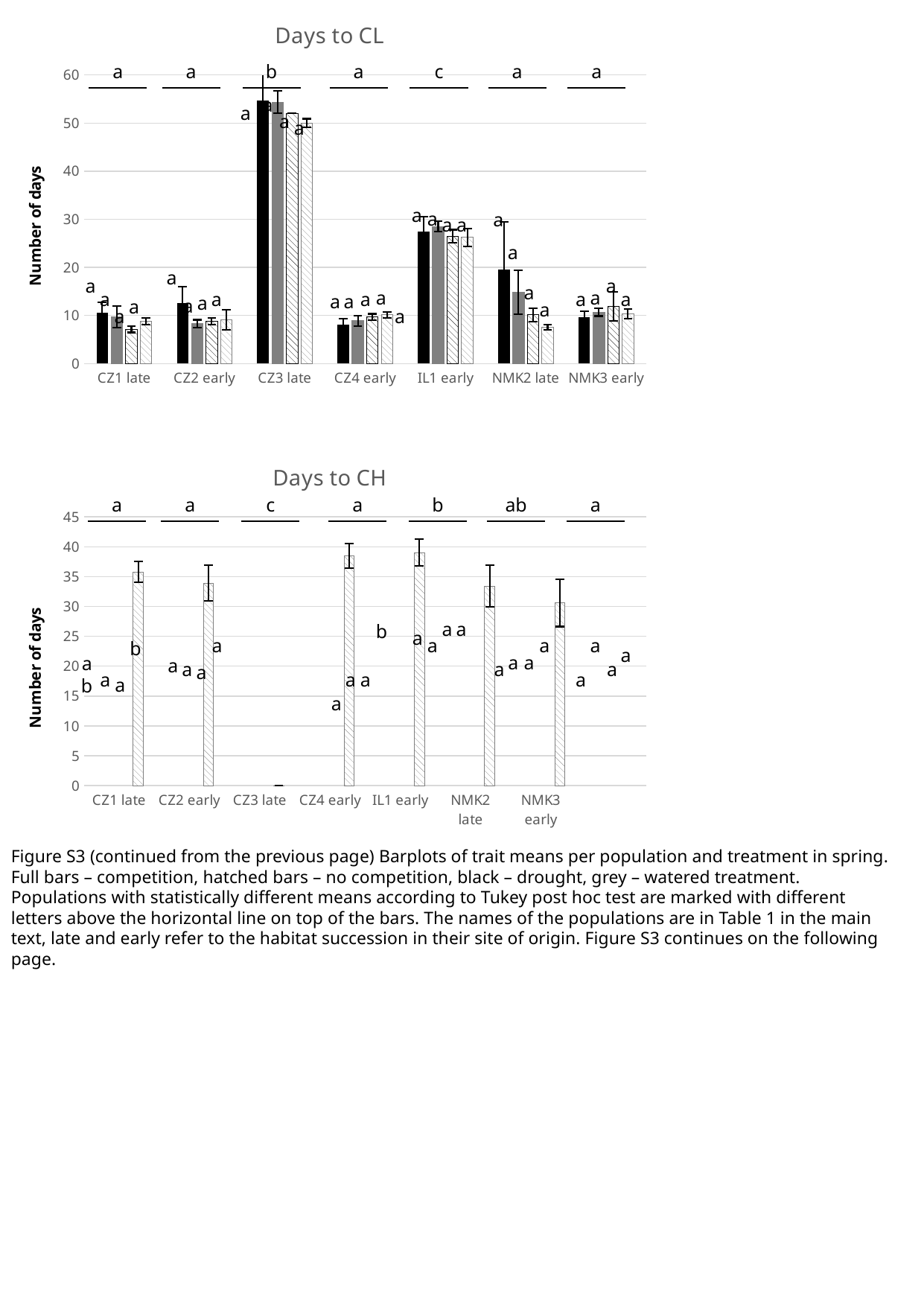

### Chart: Days to CL
| Category | axes | axes | axes | axes |
|---|---|---|---|---|
| CZ1 late | 10.55555556 | 9.75 | 7.125 | 8.777777778 |
| CZ2 early | 12.625 | 8.25 | 8.777777778 | 9.1 |
| CZ3 late | 54.71428571 | 54.33333333 | 52.0 | 50.0 |
| CZ4 early | 8.125 | 8.888888889 | 9.666666667 | 10.11111111 |
| IL1 early | 27.375 | 28.5 | 26.5 | 26.22222222 |
| NMK2 late | 19.57142857 | 14.85714286 | 10.14285714 | 7.571428571 |
| NMK3 early | 9.666666667 | 10.66666667 | 11.88888889 | 10.33333333 |a
b
a
c
a
a
a
a
a
a
a
a
a
a
a
a
a
a
a
a
a
a
a
a
a
a
a
a
a
a
a
a
a
a
a
a
### Chart: Days to CH
| Category | axes | axes | axes | axes |
|---|---|---|---|---|
| CZ1 late | 32.0 | 27.14285714 | 26.0 | 35.77777778 |
| CZ2 early | 28.0 | 30.125 | 28.0 | 33.9 |
| CZ3 late | 69.0 | 0.0 | 69.0 | 0.0 |
| CZ4 early | 18.5 | 25.85714286 | 25.125 | 38.44444444 |
| IL1 early | 36.83333333 | 35.75 | 38.25 | 39.0 |
| NMK2 late | 32.0 | 32.6 | 28.71428571 | 33.42857143 |
| NMK3 early | 25.4 | 37.0 | 28.66666667 | 30.625 |a
c
a
b
ab
a
a
a
a
b
a
a
a
a
a
b
a
a
a
ab
a
a
a
a
a
a
a
a
a
a
a
Figure S3 (continued from the previous page) Barplots of trait means per population and treatment in spring. Full bars – competition, hatched bars – no competition, black – drought, grey – watered treatment. Populations with statistically different means according to Tukey post hoc test are marked with different letters above the horizontal line on top of the bars. The names of the populations are in Table 1 in the main text, late and early refer to the habitat succession in their site of origin. Figure S3 continues on the following page.

## Slide 7
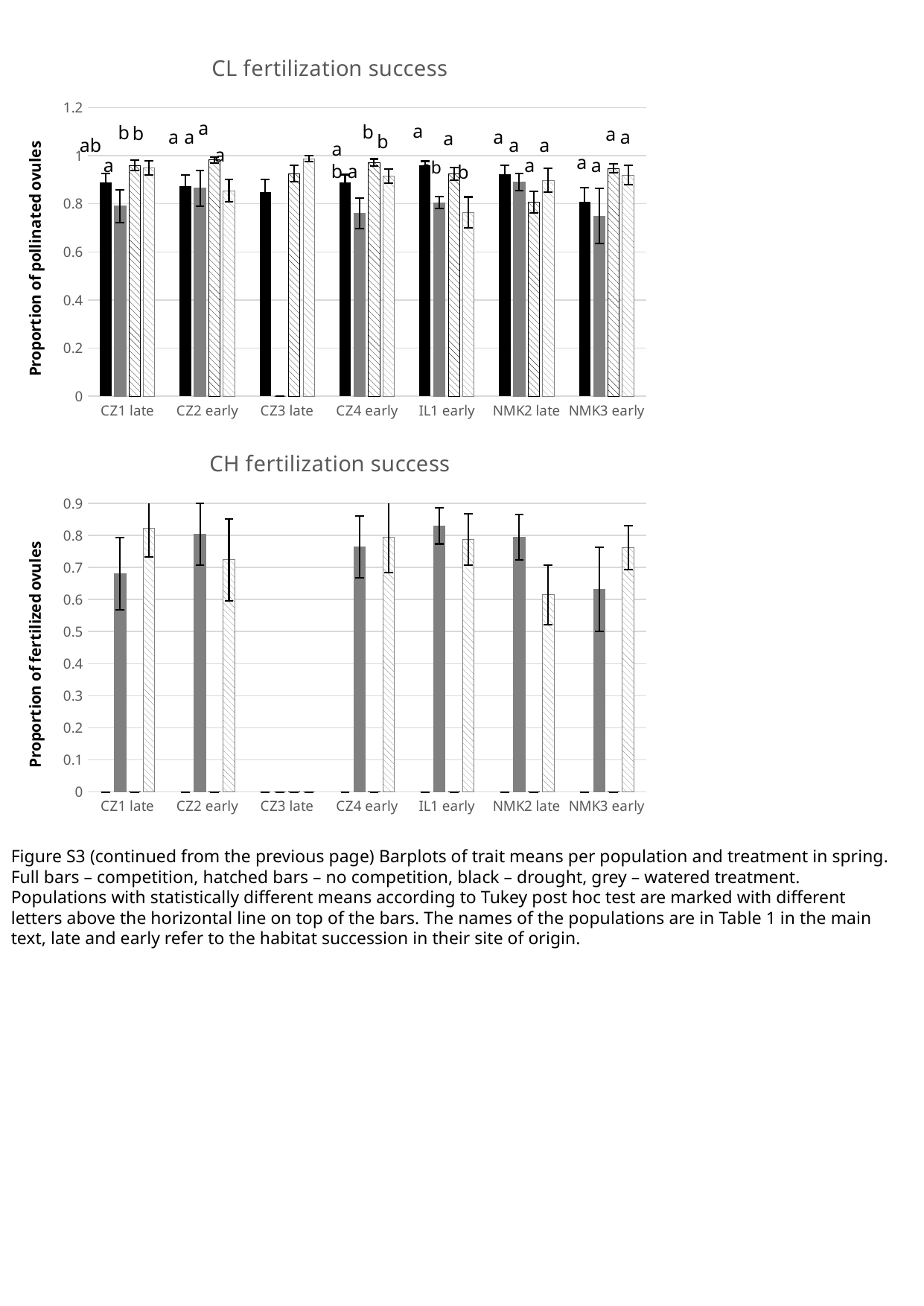

### Chart: CL fertilization success
| Category | axes | axes | axes | axes |
|---|---|---|---|---|
| CZ1 late | 0.888888889 | 0.79 | 0.96 | 0.95 |
| CZ2 early | 0.875 | 0.865 | 0.982222222 | 0.854 |
| CZ3 late | 0.848 | 0.0 | 0.926 | 0.988 |
| CZ4 early | 0.88875 | 0.761111111 | 0.971111111 | 0.915555556 |
| IL1 early | 0.95875 | 0.804 | 0.925 | 0.765 |
| NMK2 late | 0.921666667 | 0.891428571 | 0.807142857 | 0.897142857 |
| NMK3 early | 0.808333333 | 0.748333333 | 0.947777778 | 0.91875 |a
a
b
b
b
a
a
a
a
a
a
b
ab
a
a
ab
a
a
a
a
a
ab
a
b
### Chart: CH fertilization success
| Category | axes | axes | axes | axes |
|---|---|---|---|---|
| CZ1 late | 0.0 | 0.68125 | 0.0 | 0.822222222 |
| CZ2 early | 0.0 | 0.80375 | 0.0 | 0.724444444 |
| CZ3 late | 0.0 | 0.0 | 0.0 | 0.0 |
| CZ4 early | 0.0 | 0.764285714 | 0.0 | 0.79375 |
| IL1 early | 0.0 | 0.83 | 0.0 | 0.786666667 |
| NMK2 late | 0.0 | 0.794 | 0.0 | 0.615 |
| NMK3 early | 0.0 | 0.631666667 | 0.0 | 0.7625 |Figure S3 (continued from the previous page) Barplots of trait means per population and treatment in spring. Full bars – competition, hatched bars – no competition, black – drought, grey – watered treatment. Populations with statistically different means according to Tukey post hoc test are marked with different letters above the horizontal line on top of the bars. The names of the populations are in Table 1 in the main text, late and early refer to the habitat succession in their site of origin.
